# Assessment methods for senior resident attainment based on a new training model

**DOI:** 10.1055/a-2462-1488

**Published:** 2024-11-26

**Authors:** Hiroki Kato, Makoto Kobayashi, Hirotaka Takeshima, Hiroshi Nakayabu, Akihiro Maruyama, Shintaro Tominaga, Hitoshi Sugiyama

**Affiliations:** 1 37036 Gastroenterology, Yokkaichi Municipal Hospital, Yokkaichi, Japan; 2 36589 Gastroenterology, Nagoya Daigaku Daigakuin Igakukei Kenkyuka Igakubu, Nagoya, Japan

We previously reported on the use of the Endoscopist & Assistant’s Simulator dry lab (EASY; Tanac Co. Ltd., Gifu, Japan) for recruiting junior residents and training senior residents in cold snare polypectomy (CSP) 1 2 ( Fig. 1 ). This study examined the time required for CSP training to assess senior residents’ achievement levels.

**Fig. 1:**
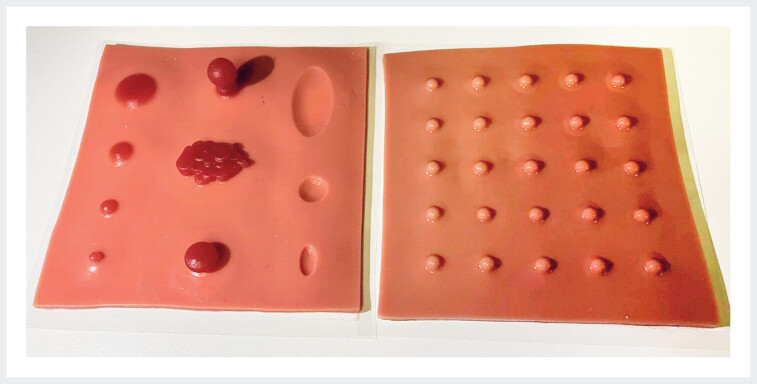
Endoscopist & Assistant’s Simulator dry lab (EASY) employed to perform resection and clip closure using a snare.

EASY, developed by Matsuzaki and Tsunemi, is an endoscopic simulator that can be used to perform resection and clip closure using a snare. This simulator was developed for convenient training in polypectomy and suturing techniques.

Two physicians in their second semester of training in our department were included as study participants. Both physicians had prior training in gastrointestinal procedures and were actively performing CSP and endoscopic mucosal resection under instructor supervision ( Video 1 ). After the physicians had completed 1 year in our department, we evaluated their CSP performance by measuring the time taken to complete CSP on 25 colorectal polyp models, assessing for successful complete resection.

Training of senior residents for cold snare polypectomy using Endoscopist & Assistant’s Simulator dry lab (EASY).Video 1

The results demonstrated increased speed compared to 1 year earlier ( Fig. 2 , Fig. 3 , Fig. 4 ). This improvement is likely attributable to the training conducted at the hospital. For trainees, comparing themselves with an instructor can set an unrealistically high benchmark. However, by tracking their current performance against previous achievements, trainees can objectively measure their improvement, which can be highly motivating.

**Fig. 2:**
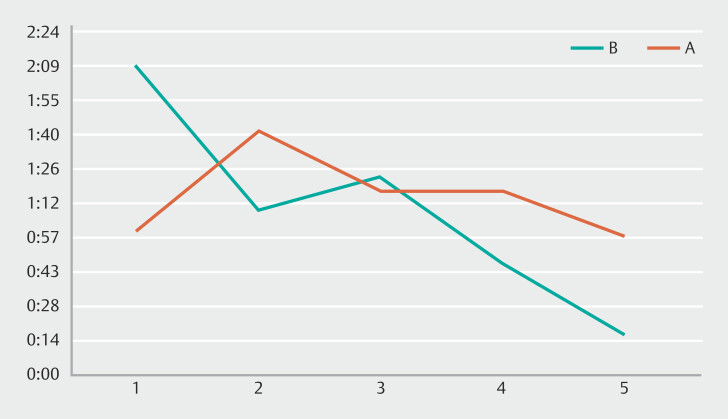
Time taken by Trainees A (red) and B (green) for the first attempt of cold snare polypectomy.

**Fig. 3:**
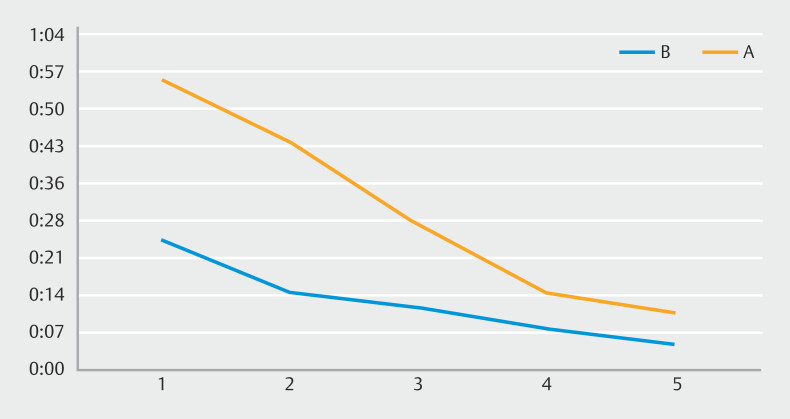
Time taken by Trainees A (orange) and B (blue) for the second attempt of cold snare polypectomy.

**Fig. 4:**
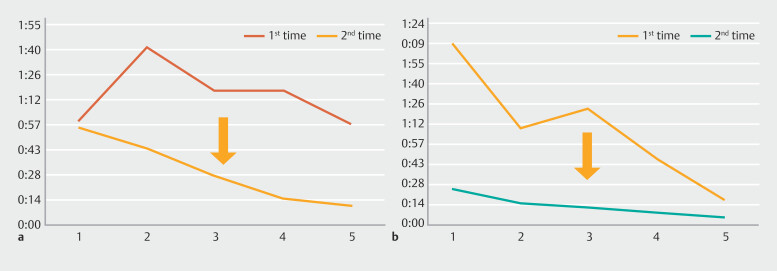
Time taken for the first and second attempts of cold snare polypectomy by Trainees A (red and orange, respectively) and B (green and blue, respectively).

Time-based CSP training using EASY is an effective motivational tool, as it allows trainees to objectively assess their progress over time.

Endoscopy_UCTN_Code_TTT_1AU_2AB

## Notes

Endoscopy E-Videos https://eref.thieme.de/e-videos
*E-Videos* is an open access online section of the journal *Endoscopy* , reporting on interesting cases and new techniques in gastroenterological endoscopy. All papers include a high-quality video and are published with a Creative Commons CC-BY license. Endoscopy E-Videos qualify for HINARI discounts and waivers and eligibility is automatically checked during the submission process. We grant 100% waivers to articles whose corresponding authors are based in Group A countries and 50% waivers to those who are based in Group B countries as classified by Research4Life (see: https://www.research4life.org/access/eligibility/ ). This section has its own submission website at https://mc.manuscriptcentral.com/e-videos .
